# Supplementary material for: Histidine is selectively required for the growth of Myc‐dependent dedifferentiation tumours in the Drosophila CNS
Source: EMBO J. 2019 Feb 25;38(7):e99895. doi: 10.15252/embj.201899895 (PMC6443203; doi:10.15252/embj.201899895)
Supplement: Supplementary file 1 — Expanded View Figures PDF [file EMBJ-38-e99895-s001.pdf]

Expanded View Figures

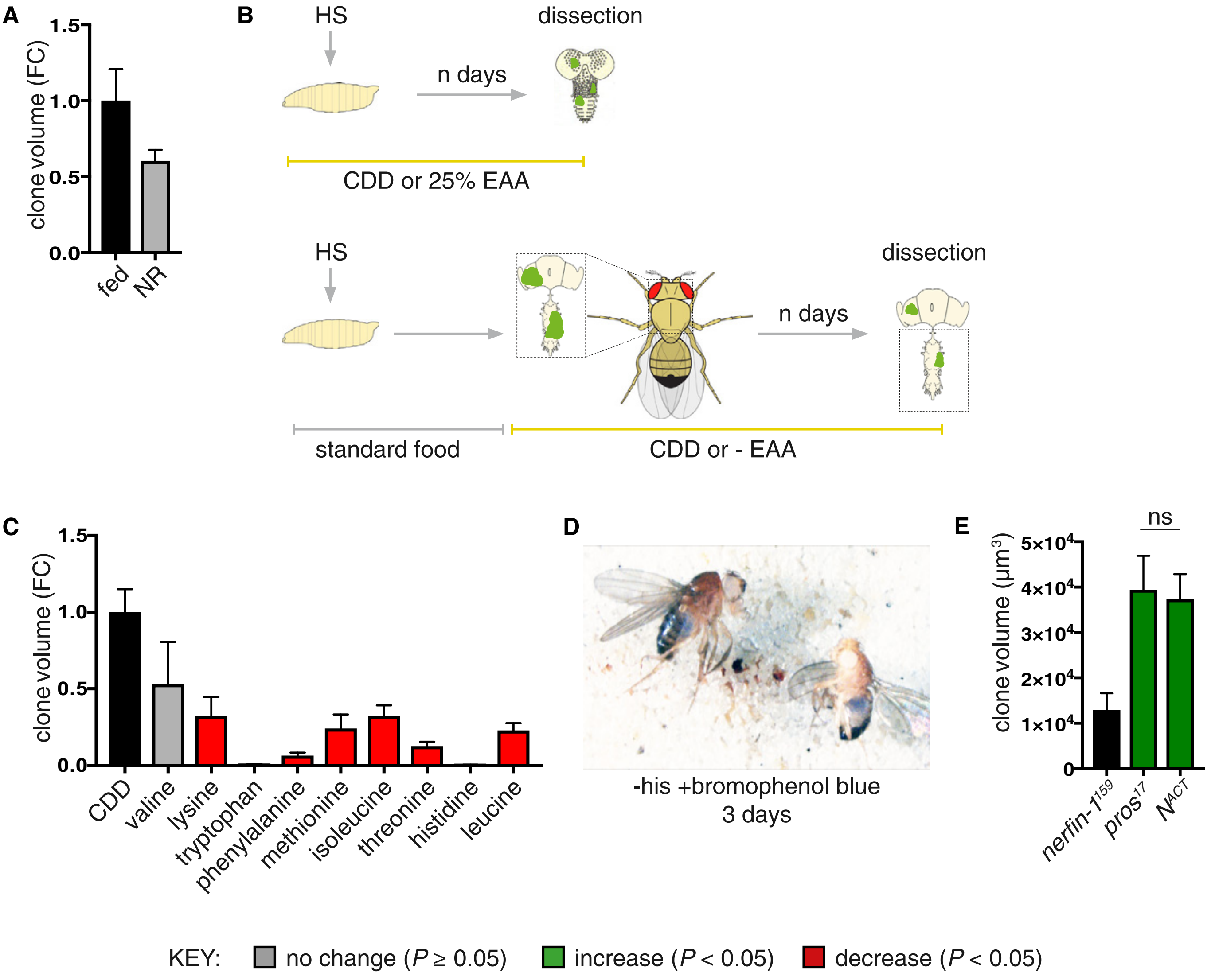

Figure EV1. The effect of nutrient restriction and EAA withdrawal on *nerfin-1* mutant clone growth (related to Fig 1).

A *nerfin-1* clones were induced at 48 h ALH and transferred to standard *Drosophila* media or agarose/PBS (nutrient restriction, NR) for 3 days, and clone size is reduced (though not significantly, P = 0.054) under NR (n = 90, 116).

B Schematic depicting heat shock and dissection regimes for larval and adult EAA dietary manipulations.

C Volume (fold change) of the sum of GFP$^+$ *nerfin-1* clones per CNS in animals where clones were induced at 48 h ALH for 1 h and evaluated after 9 days of feeding on CDD or CDD-EAAs (n = 17, 3, 8, 5, 12, 5, 10, 10, 17, 9).

D w^{118} adults (left, female, right, male) were fed a —his diet labelled with bromophenol blue; blue food was detected in the gut after 3 days of feeding.

E Larval *nerfin-1, pros* and N^{ACT} clone volume, clones were induced at 48 h ALH and measured at 6 days ALH (n = 26, 11, 16). *pros* and N^{ACT} are of comparable volume, ns = not significant.

Data information: In all graphs, the key indicates that green bars represent a significant increase (P < 0.05), red bars a significant decrease (P < 0.05), and grey bars no significant change (P > 0.05) in *t*-tests with the relevant paired controls (black bar). In all graphs, error bars represent 1 standard error of the mean (SEM). FC, fold change.

© 2019 The Authors

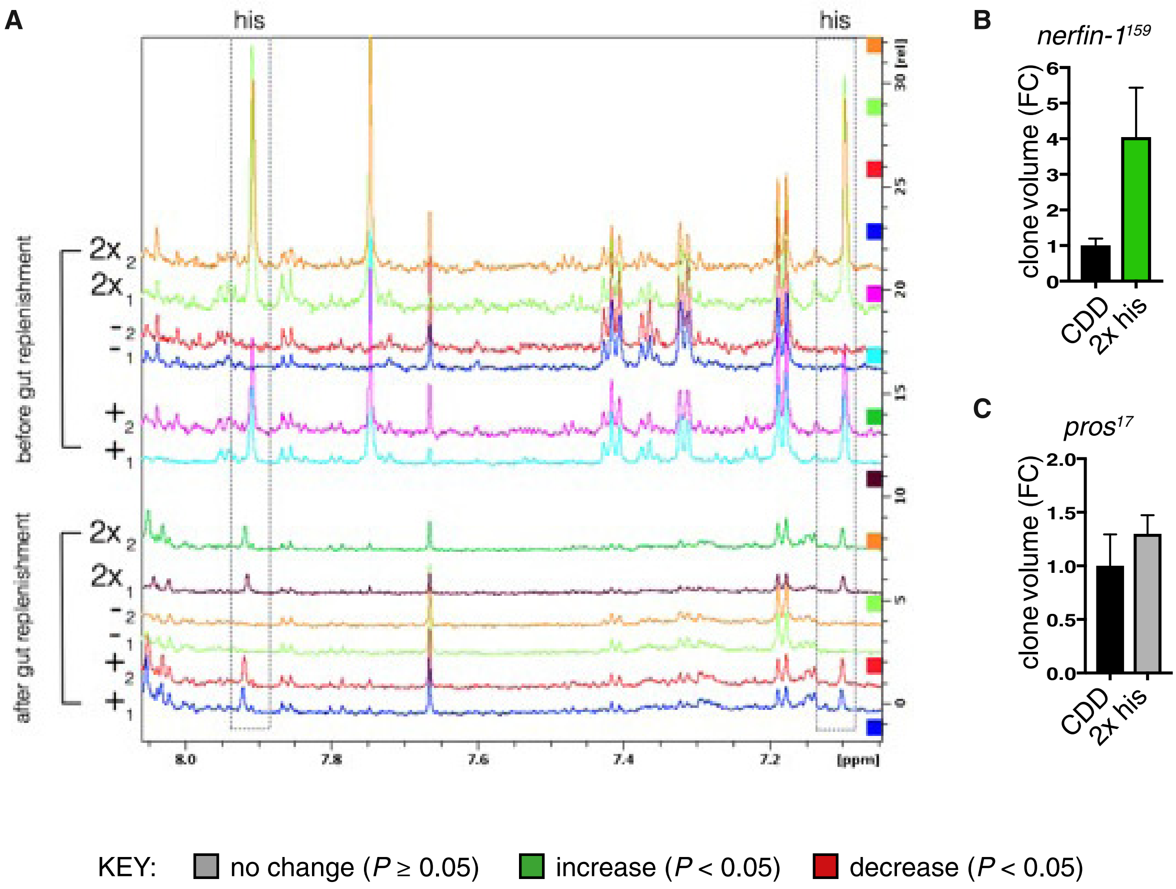

Figure EV2. ^{1}H NMR can detect changes in dietary histidine and his dietary manipulation differentially affects *nerfin-1*$^-$ and *pros*$^-$ clonal growth (related to Fig 2).

A Spectra showing a region from 8.0 to 7.1 ppm from extracted whole adult fly polar metabolome before (upper panel) and after (lower panel) clearance of dietary histidine from the gut (see Materials and Methods), boxed panel highlights the histidine peak. Replica peaks for histidine are seen in the boxed areas at ~7.9 (ε-proton) and 7.1 (δ-proton) ppm in histidine-replete: "+" (0.5 g/l his) and "2x" (1.0 g/l his) CDD profiles; histidine peaks are not seen in "–" (0 g/l his) profiles in either panel.

B *nerfin-1*$^-$ clone volume is increased by ~4-fold upon feeding on 2× his (n = 24, 15).

C *pros*$^-$ clone volume did not significantly changed upon feeding on 2× his (n = 19, 34).

Data information: In all graphs, the key indicates that green bars represent a significant increase ($P < 0.05$), red bars a significant decrease ($P < 0.05$), and grey bars no significant change ($P > 0.05$) in t-tests with the relevant paired controls (black bar). In all graphs, error bars represent 1 standard error of the mean (SEM). FC, fold change.

 © 2019 The Authors

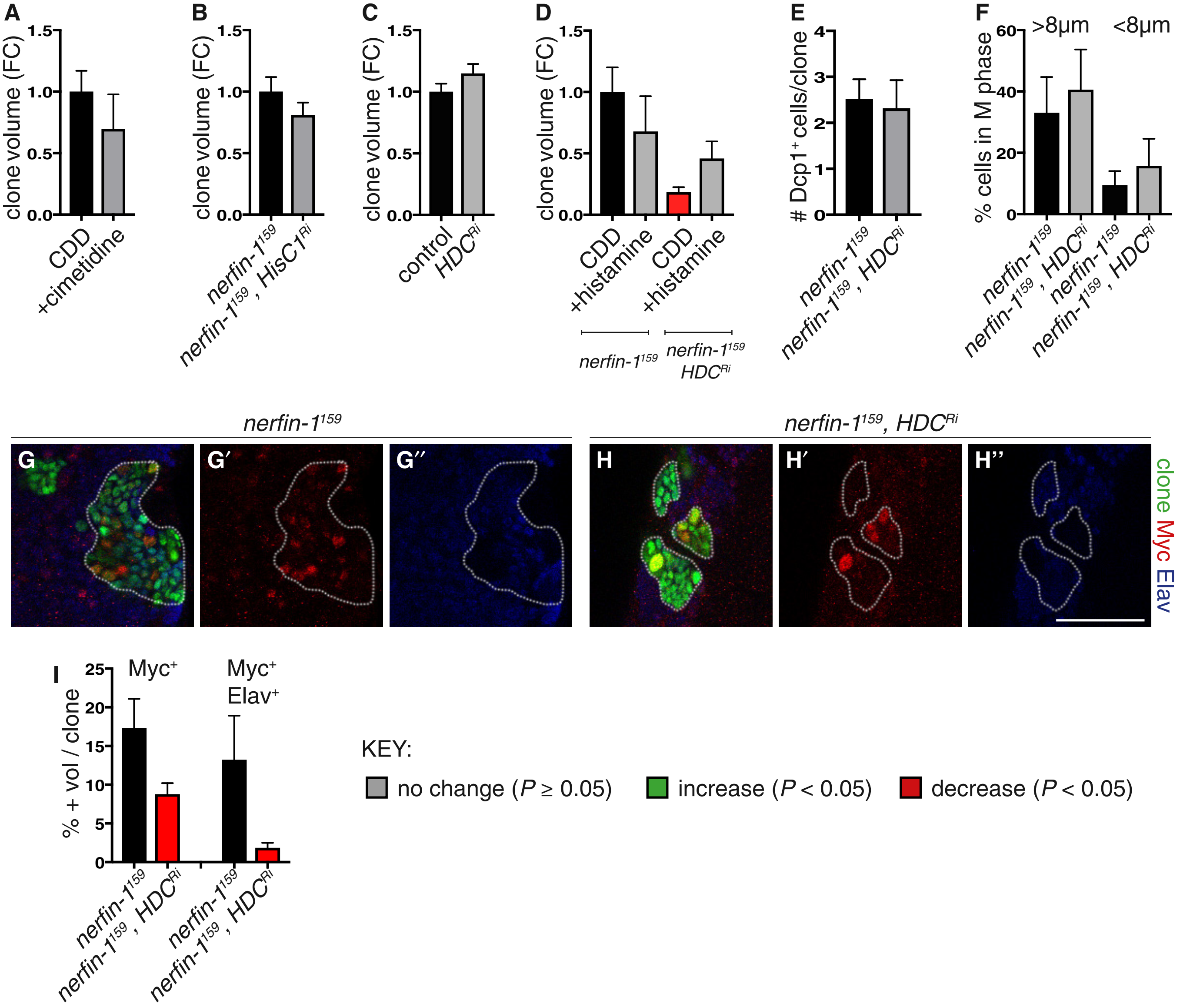

Figure EV3. The effect of manipulating histidine metabolic pathway intermediates on control and *nerfin-1* clones (related to Figs 3 and 4).

A 500 mg/ml of the histamine receptor inhibitor cimetidine (n = 39, 23) did not significantly alter *nerfin-1* clonal growth in the adult CNS.

B Knockdown of histamine inhibitor HisCl1 ($HisCl1^{Ri}$), knockdown verified in Oh *et al*, 2013, did not significantly alter *nerfin-1* clonal growth in the larval CNS (n = 37, 42).

C Hdc knockdown did not significantly alter the growth of wildtype larval CNS clones (n = 42, 36).

D Histamine supplementation did not significantly increase *nerfin-1* clone size, but significantly rescued *nerfin-1;HdcRi* clonal growth in the larval CNS (n = 15, 10, 12, 9).

E Hdc knockdown did not significantly alter the amount of cell death in *nerfin-1* clones in the larvae (n = 43, 35).

F The speed of the cell cycle in fully dedifferentiated NBs > 8 µm (n = 11, 9) and dedifferentiating neurons < 8 µm (n = 11, 9) was not significantly altered by Hdc inhibition in *nerfin-1* larval clones.

G–I Representative pictures showing that Hdc inhibition significantly reduced the % of Myc^+ NBs (red) and Myc^+ $Elav^+$ (blue) neurons in *nerfin-1* larval clones (G–H”), quantified in (I) (n = 14, 14, 13, 16). Scale bar = 50 µm.

Data information: In all graphs, the key indicates that green bars represent a significant increase (P < 0.05), red bars a significant decrease (P < 0.05), and grey bars no significant change (P > 0.05) in t-tests with the relevant paired controls (black bar). In all graphs, error bars represent 1 standard error of the mean (SEM). FC, fold change.

© 2019 The Authors

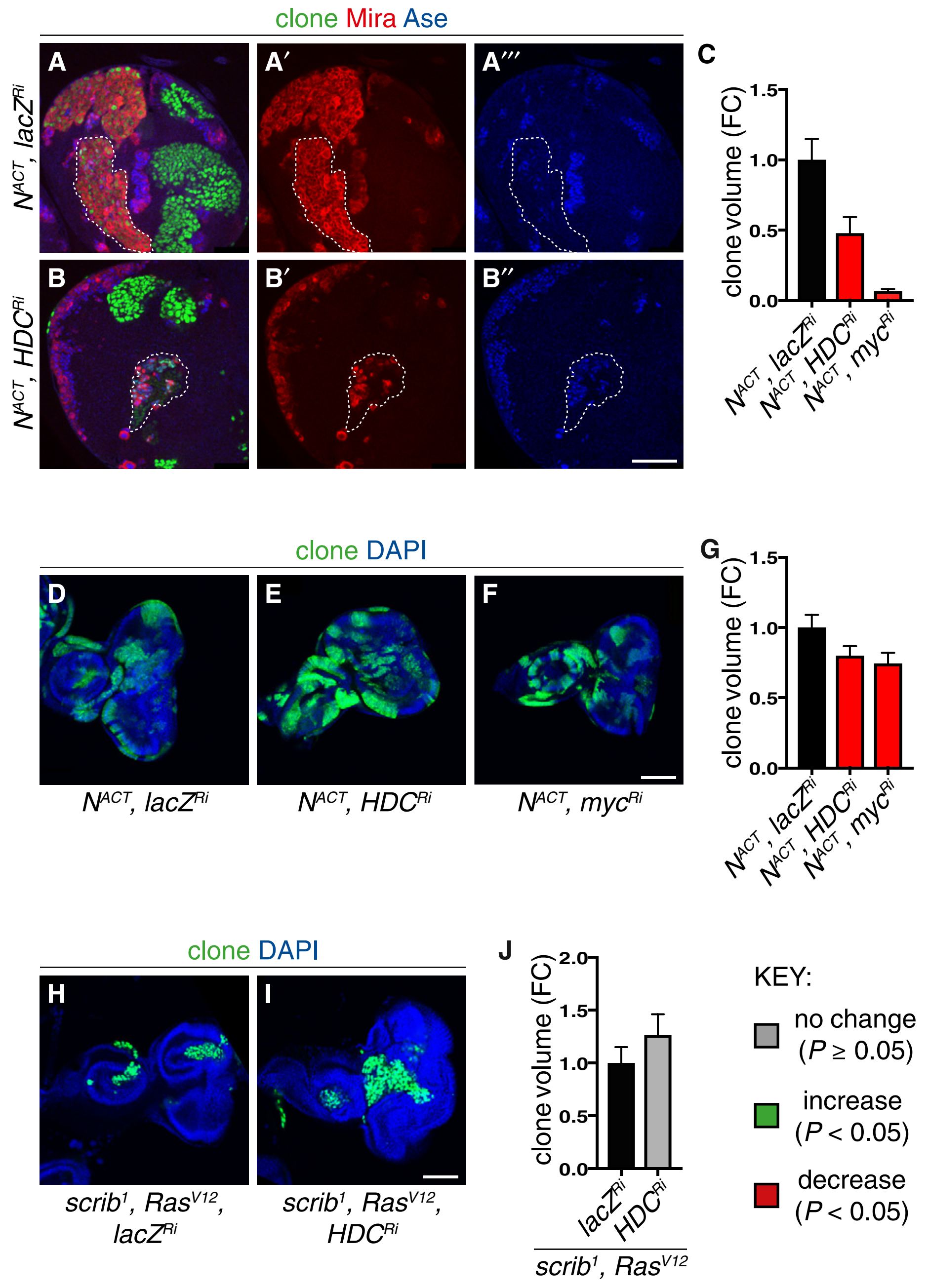

Figure EV4. Hdc knockdown reduces N^{ACT} larval type II CNS clones, but not the growth of N^{ACT} and *scrib*^{1};*Ras*^{V12} larval eye imaginal disc clones (related to Figs 1 and 4).

A–C Representative pictures showing that larval type II N^{ACT} MARCM clonal growth was significantly reduced by Hdc (A–B”) and Myc knockdown, quantified in (C) (*n* = 11, 7, 7). Scale bar = 50 μm.

D–G Representative pictures showing that Myc (F) and Hdc (E) inhibition was sufficient to significantly reduce N^{ACT} clonal growth in the larval eye imaginal epithelia, quantified in (G) (*n* = 75, 80, 75). Scale bar = 100 μm.

H–J Hdc inhibition did not significantly alter the growth of *scrib*^{1};*Ras*^{V12} larval eye imaginal epithelia clones, quantified in (J) (*n* = 57, 33). Scale bar = 100 μm.

Data information: In all graphs, the key indicates that green bars represent a significant increase ($P < 0.05$), red bars a significant decrease ($P < 0.05$), and grey bars no significant change ($P > 0.05$) in *t*-tests with the relevant paired controls (black bar). In all graphs, error bars represent 1 standard error of the mean (SEM). FC, fold change.

© 2019 The Authors
